# PN3b as an independent risk factor for poor prognosis and peritoneal recurrence in Borrmann type IV gastric cancer: A retrospective cohort study

**DOI:** 10.3389/fsurg.2022.986696

**Published:** 2022-11-10

**Authors:** Yiran Chen, Yanyan Chen, Liping Wen, Laizhen Tou, Haiyong Wang, Lisong Teng

**Affiliations:** ^1^ Department of Surgical Oncology, The First Affiliated Hospital, College of Medicine, Zhejiang University, Hangzhou, China; ^2^ Department of Gastrointestinal Surgery, Lishui Hospital, College of Medicine, Zhejiang University, Lishui, China

**Keywords:** gastric cancer, peritoneal metastasis, lymph node metastasis, Borrmann type, signet ring cell carcinoma

## Abstract

**Background:**

The clinicopathological features and surgical treatment strategies of Borrmann type IV gastric cancer (GC) remain controversial. Peritoneal metastasis is the most common recurrence pattern in patients with Borrmann type IV GC.

**Methods:**

Among 2026 gastric cancer between January 2009 and August 2019, 159 cases of Borrmann type IV GC were included in this study (7.8%). We retrospectively analyzed the clinicopathological characteristics and prognosis of these patients. Univariate and multivariate Cox proportional hazards were applied to identify independent prognostic factors. Predictors related to peritoneal metastasis of type IV GC were analyzed by multivariate Cox regression analysis.

**Results:**

Borrmann type IV gastric cancer was associated with more advanced clinicopathological features at diagnosis than the other Borrmann type GC. Of the 159 patients with Borrmann type IV GC, the median OS was 23 months. The number of patients with peritoneal metastasis was 43, accounted for 27.0% of all the patients and 87.8% of the patients with distant metastasis. Multivariate analyses revealed lymph node metastasis to be independent prognostic factor for survival in Borrmann type IV GC patients. pN3b and tumor size > 50 mm showed to be risk factors for peritoneal metastasis.

**Conclusions:**

Borrmann type IV GC is an important independent prognostic factor. pN3b is an independent prognostic factor and a predictor of peritoneal metastasis in patients with Borrmann type IV GC.

## Introduction

Gastric cancer (GC) is one of the most common malignancies in the world, with more than 1.8 million new cases worldwide in 2020 and an estimated 770,000 deaths, making it the fifth most frequently diagnosed cancer and the fourth in mortality (1). The Borrmann type proposed in 1926 provides a relatively accurate description of the gross morphology of advanced gastric cancer (2), among which Borrmann type IV GC accounts for about 8%–13% (3–6). Borrmann type IV GC, including linitis plastica, are characterized by poorly differentiated tumor cells with diffusely infiltrative involvement of the stomach (5, 7, 8). The patients were frequently associated with poor tumor differentiation, lymph node metastases, peritoneal metastases, serosal invasion, lymphatic invasion, and poor prognosis (4, 9–11). Peritoneal metastasis (PM) represents the most common type of recurrence in advanced GC and is considered as an independent factor for poor prognosis (12). Although the treatments of peritoneal metastasis in gastric cancer have made some progress, its prognosis is still poor (13, 14). Early detection and intervention are still the main way to prolong the life of patients. Lee et al. reported that Borrmann type IV gastric cancer is an independent risk factor for peritoneal recurrence (15). However, in Borrmann type IV GC, the risk factors of peritoneal recurrence have not been well studied. Prognostic factors have the potential to play an important role in improving health, including clinical practice, healthcare research, and the development, evaluation, and targeting of interventions (16). Therefore, in our study, we described the clinicopathological features of Borrmann type IV gastric cancer and focus on the risk factors for PM in this special type of gastric cancer.

## Material and methods

### Probands

**Figure 1:**
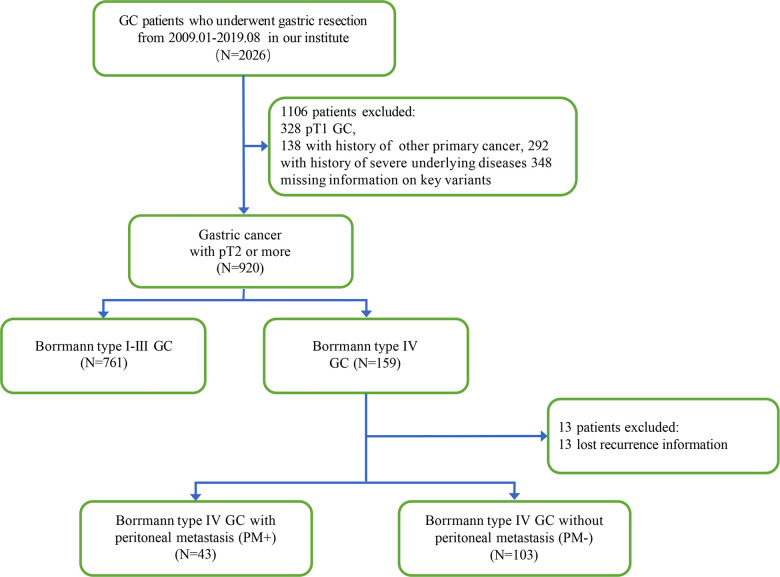
Study workflow diagram (GC, gastric cancer; PM, peritoneal metastasis).

Between January 2009 and August 2019, 2026 gastric cancer patients underwent gastric resection at the department of surgical oncology, the First Affiliated Hospital, Zhejiang University. Among these patients, 159 cases of Borrmann type IV GC were included in further analysis (7.8%). In addition, 761 cases of Borrmann type I–III GC were selected for comparison with Borrmann type IV GC. The inclusion criteria for patients were as follows: (i) patients diagnosed with gastric cancer with pT2 or more from January 2009 to August 2019; (ii) patients received radical gastrectomy or palliative gastrectomy in the department of surgical oncology; and (iii) patients had complete clinical data and pathologic specimens available for reevaluation. The exclusion criteria for patients included: (i) patients diagnosed with combined primary malignant cancer; (ii) patients had history of severe underlying diseases; and (iii) missing information for key variants. The specific patient selection pathway is shown in Figure 1. Each patient was consented to collecting research data once the hospital file is created. Outcomes of our interest, that is, overall survival (OS) and time to first recurrence (RFS), were collected during the follow-up period (median follow-up time 38 months, ranging from 1 month to 122 month). 18 Borrmann type IV GC patients were lost in post-operative follow-up. Age, gender, CEA and CA19-9 level before surgery, surgical intervention, pathological features including histological type, tumor location, tumor size, depth of tumor invasion and lymph node metastasis, were retrospectively collected from the medical record system of our institution. The tumors were staged according to the eighth edition of AJCC/UICC TNM staging system.

### Statistical analysis

The clinicopathological features between Borrmann type IV GC and Borrmann type I–III GC were compared using Chi-square or Fisher's exact test. Log-rank tests were used to analyze survival curves which were created using Kaplan-Meier analysis. Information obtained from the univariate analysis was applied to a survival analysis with covariates using the Cox model of proportional hazards (forward likelihood ratio model). Subsequently, Cox proportional hazard regression (enter model) was used to examine the effect of different clinicopathological features and treatment on PM in patients with Borrmann type IV gastric cancer and predict the independent risk factors of PM in such patients. Statistical analysis was performed using SPSS 26.0 software. In all statistical analyses, *P* < 0.05 was considered significant.

## Results

### Characteristics of Borrmann type IV GC patients

Borrmann type IV GC patients showed significant differences in the distribution of gender, operation type, surgical curability, tumor size, differentiation, depth of invasion, lymph node metastasis, distant metastasis and stage comparing with other Borrmann types of gastric cancer (Supplementary Table S1). Multivariate analysis showed that Borrmann type IV GC was an independent prognostic factor (Supplementary Figures S1, S2 and Supplementary Table S2) after adjusting for age, gender, residual tumor, differentiation, TNM stage, serum CEA and CA19-9 level. The clinicopathological features of Borrmann type IV GC patients are shown in Table 1. Of 159 patients with Borrmann type IV GC who underwent gastrectomy, 89 (56.0%) were male and 70 (44.0%) were female. The mean age was 59-year-old (range 16 to 87). Total gastrectomy was performed in 97 patients (61.0%) and subtotal gastrectomy was performed in 62 patients (39.0%). D2/D2 + lymphadenectomy was applied to all stage I–III patients. 7 patients received D2 lymph node dissection including splenectomy because of tumor invasion. The average number of lymph nodes examined was 26.8. Among the 26 patients diagnosed as stage IV, 8 patients received palliative resection due to gastrointestinal bleeding or obstruction and others were found to have distant metastasis during the operation or post-operative pathology confirmed distant metastasis. 12 cases (7.6%) underwent extended surgical resection due to tumor invasion or concurrent indications for resection. Among these patients, 115 patients received at least 1 cycle of first-line chemotherapy (platinum- or taxane-based), in which 25 patients received neoadjuvant chemotherapy and 109 patients received adjuvant chemotherapy. The purpose of these patients receiving chemotherapy included not only preoperative drawdown and postoperative adjuvant, but also conversion chemotherapy.

**Table 1:** Clinicopathological features and survival analysis of patients with Borrmann type IV gastric cancer.

Factors	Borrmann IV GC (*n* = 159)	Median OS (month)	Hazard Ratio (95% CI)	*P* Value
Age	16–87 (59)			
**Gender**				
Female	70 (44.0%)	24	Reference	
Male	89 (56.0%)	21	1.166 (0.746–1.822)	0.494
**Operation**				
Distal gastrectomy	55 (34.6%)	49	Reference	0.029
Total gastrectomy	97 (61.0%)	18	2.341 (1.388–3.947)	
Proximal gastrectomy	2 (1.3%)	1	9.124 (2.079–40.041)	
Residual gastrectomy	5 (3.1%)	–	0.676 (0.090–5.067)	
**Residual Tumor**				
R0	123 (77.4%)	32	Reference	<0.001
R1^#^	20 (12.6%)	16	2.273 (1.280–4.034)	
R2	11 (6.9%)	7	3.368 (1.518–7.472)	
NA	5 (3.1%)	–	–	
**Tumor Size**				
≤50 mm	57 (35.8%)	47	Reference	<0.001
>50 mm	102 (64.2%)	18	2.458 (1.468–4.113)	
**Tumor Location**				
Upper 1/3	10 (6.3%)	13	Reference	0.003
Upper-middle	7 (4.4%)	26	0.315 (0.067–1.487)	
Middle 1/3	35 (22.0%)	23	0.516 (0.219–1.219)	
Middle-lower	18 (11.3%)	19	0.741 (0.297–1.845)	
Lower 1/3	55 (34.6%)	35	0.496 (0.222–1.107)	
Entire	29 (18.2%)	10	1.580 (0.692–3.606)	
Residual stomach	5 (3.1%)	–	0.254 (0.032–2.039)	
**Depth of Invasion**				
T2	8 (5.0%)	–	Reference	0.071
T3	50 (31.4%)	26	6.026 (0.810–44.840)	
T4a	75 (47.2%)	20	8.056 (1.108–58.566)	
T4b	26 (16.4%)	17	7.999 (1.036–61.761)	
**Lymph Node Metastasis**				
N0	19 (11.9%)	47	Reference	<0.001
N1	17 (10.7%)	49	1.004 (0.251–4.015)	
N2	29 (18.2%)	26	2.888 (0.971–8.593)	
N3a	43 (27.0%)	27	2.777 (0.959–8.037)	
N3b	51 (32.1%)	14	5.732 (2.010–16.348)	
**Distant Metastasis**				
M0	133 (83.6%)	29	Reference	<0.001
M1	26 (16.4%)	6	3.684 (2.178–6.232)	
**TNM Stage**				
I	5 (3.1%)	–	–	<0.001
II	22 (13.8%)	47	Reference	
III	106 (66.6%)	23	3.030 (1.212–7.574)	
IV	26 (16.4%)	6	9.292 (3.436–25.127)	
**CEA**				
≤5.0 ng/ml	109 (68.6%)	24	Reference	0.038
>5 ng/ml	32 (20.1%)	13	1.720 (1.019–2.906)	
NA	18 (11.3%)	–	–	
**CA19-9**				
≤37 U/ml	106 (67.3%)	26	Reference	0.002
>37 U/ml	34 (21.4%)	12	2.167 (1.300–3.612)	
NA	18 (11.3%)	–	–	
**Chemotherapy**				
No	38 (23.9%)	16	Reference	0.035
Yes*	115 (72.3%)	29	0.591 (0.362–0.964)	
NA	6 (3.8%)	–	–	

Abbreviations: NA, not available.

^#^ R1, postoperative pathology showed positive surgical margin.

* Including the patients received neoadjuvant chemotherapy (*n* = 25) and adjuvant chemotherapy (*n* = 109).

### Prognostic significance of Borrmann type IV GC

**Figure 2:**
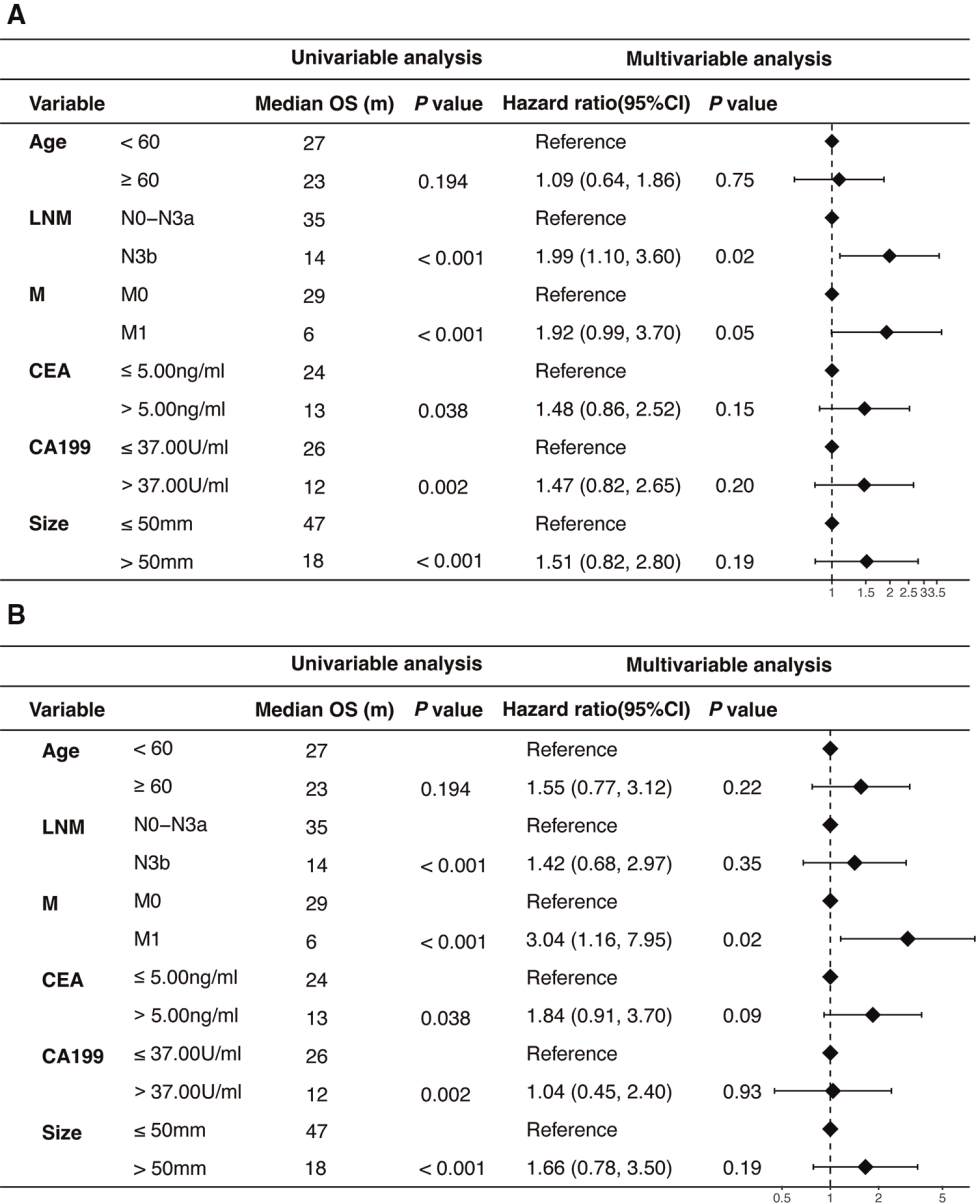
Cox proportional hazard regression models and forest plot for overall survival. (**A**) Forest plot displaying the results of hazard ratio for overall survival of patients with Borrmann type IV gastric cancer; (**B**) Forest plot displaying the results of hazard ratio for overall survival of patients with B-4 GC after receiving R0 resection. (LNM, lymph node metastasis; M, distant metastasis; CI, confidence interval).

**Figure 3:**
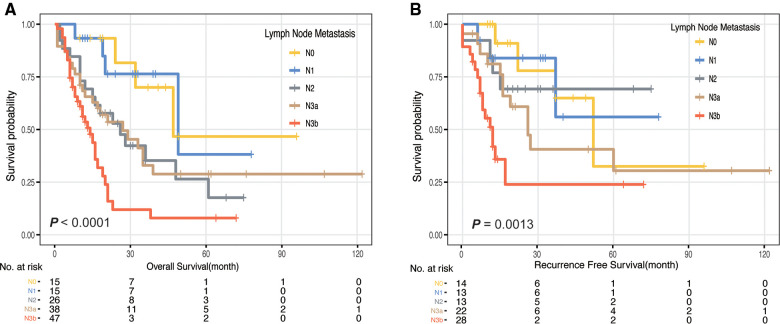
Kaplan-Meier curve of pN stage. (**A**) Kaplan-Meier curves of overall survival (OS) according to N category in Borrmann type IV gastric cancer. (**B**) Kaplan-Meier curves of recurrence-free survival (RFS) according to N category in Borrmann type IV gastric cancer.

The median OS of patients with Borrmann type IV GC was 23 months, and the 5-year survival rate was 25.1% (Supplementary Figure S3). Univariate Cox analysis revealed that residual tumor, tumor size, lymph node metastasis, distant metastasis, serum CEA and CA19-9 level were significantly associated with OS (Table 1 and Supplementary Figures S4A–F). Multivariate analysis showed that N category and distant metastasis were the independent prognostic factors (Figure 2A) after adjusting for age, CEA, CA199 and tumor size. As for patients who received R0 resection, only distant metastasis was the independent prognostic factors after adjusting for age, lymph node metastasis, CEA, CA199 and tumor size (Figure 2B). Notably, pN3b was associated with the worst prognosis, which was significantly worse than pN3a (Table 1 and Figure 3A).

### Risk factors for peritoneal metastasis of Borrmann type IV GC

43 patients developed PM, accounting for 27% of all patients and 87.8% of the patients with distant metastasis. Among them, 23 patients had synchronous PM and 20 developed metachronous PM (peritoneal recurrence). The median OS of patients with PM was significantly shorter than that of patients without PM (16 months vs. 29 months, *P* = 0.001, Supplementary Figure S5). The 3-year survival rate was 12.7%. 1-year survival rate of patients with synchronous PM was 22.5% and that of patients who develop metachronous PM (peritoneal recurrence) was 77.4%. The median OS after metachronous PM was 11 months. Among 159 patients with Borrmann type IV GC, 103 patients were followed up to date or died without peritoneal recurrence or metastasis. To investigate the risk factors for PM in patients with Borrmann type IV GC, we selected these 103 patients and 43 patients with PM for comparative analysis. The clinicopathological features are presented in Table 2. Through univariate Cox analysis, we found that there were significant differences in the prognosis and recurrence risk between pN3b and other N categories (Figures 3A,B). Therefore, when discussing the risk factors for peritoneal recurrence, we divided the N category into N0-3a and N3b with lymph node metastasis >15 as cut-off value.

**Table 2:** Clinicopathological features between PM (+) and PM (−) patients with Borrmann type IV gastric cancer.

Factors	PM (−) (*n* = 103)	PM (+) (*n* = 43)	Odds Ratio (95%CI)	*P* value
**Gender**				
Female	45 (72.6%)	17 (27.4%)	Reference	
Male	58 (69.0%)	26 (31.0%)	0.963 (0.474–1.958)	0.917
**Age**				
<60	45 (64.3%)	25 (35.7%)	Reference	
≥60	58 (76.3%)	18 (23.7%)	0.613 (0.293–1.283)	0.194
**Signet ring cell**				
No	78 (70.9%)	32 (29.1%)	Reference	
Yes	25 (69.4%)	11 (30.6%)	1.417 (0.651–3.084)	0.380
**CEA**				
≤5.0 ng/ml	71 (71.0%)	29 (29.0%)	Reference	
>5.0 ng/ml	21 (72.4%)	8 (27.6%)	1.341 (0.506–3.557)	0.555
NA	11	6		
**Depth of Invasion**				
T2–3	42 (76.4%)	13 (23.6%)	Reference	
T4	61 (67.0%)	30 (33.0%)	1.486 (0.712–3.102)	0.292
**Residual Tumor**				
R0	85 (75.9%)	27 (24.1%)	Reference	
R1^#^	11 (57.9%)	8 (42.1%)	3.145 (1.297–7.654)	
R2	3 (30.0%)	7 (70.0%)	8.534 (3.052–23.865)	<0.001^a^
**Lymph Node Metastasis**				
N0-3a	75 (75.8%)	24 (24.2%)	Reference	
N3b	28 (59.6%)	19 (40.4%)	3.349 (1.610–6.966)	0.001
**Tumor Size**				
≤50 mm	43 (81.1%)	10 (18.9%)	Reference	
>50 mm	60 (64.5%)	33 (35.5%)	3.350 (1.439–7.797)	0.005
**CA19-9**				
≤37 U/ml	73 (73.0%)	27 (27.0%)	Reference	
>37 U/ml	19 (65.5%)	10 (34.5%)	2.613 (1.124–6.072)	0.026
NA	11	6		
**Chemotherapy**				
No	28 (82.4%)	6 (17.6%)	Reference	
Yes*	71 (66.4%)	36 (33.64%)	3.096 (0.736–13.027)	0.088
NA	4	1	–	

Abbreviations: PM, peritoneal metastasis; NA, not available.

^a^ Fisher's exact test, others using Pearson's Chi square test.

^#^ R1, postoperative pathology showed positive surgical margin.

* Including the patients received neoadjuvant chemotherapy (*n* = 23) and adjuvant chemotherapy (*n* = 102).

**Figure 4:**
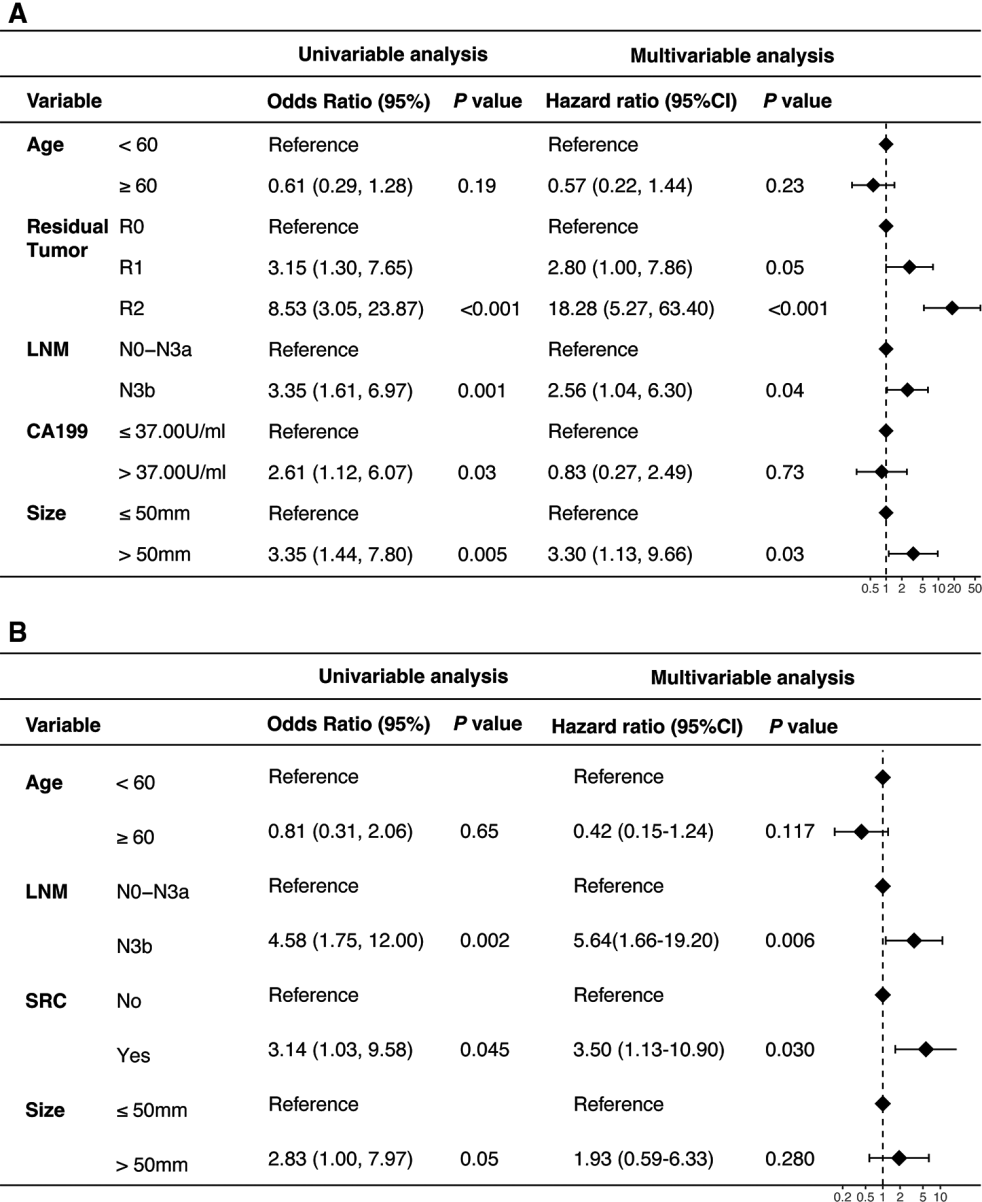
Cox proportional hazard regression models and forest plot for recurrence free survival. (**A**) Forest plot of data from multivariate cox regression revealing factors independently associated with peritoneal metastasis of patients with Borrmann type IV gastric cancer. (**B**) Forest plot of data from multivariate cox regression revealing factors independently associated with peritoneal metastasis of patients with Borrmann type IV gastric cancer after receiving R0 resection. (LNM, lymph node metastasis; SRC, signet ring cell; CI, confidence interval).

Univariate Cox analysis showed that factors related to PM were regional lymph node metastasis, tumor size, residual tumor and preoperative CA19-9 level (Table 2). Multivariate cox regression analysis revealed that pN3b (*P* = 0.04) and tumor size >50 mm (*P* = 0.03) were the independent risk factors of PM after adjusting for age and CA199 (Figure 4A). Meanwhile, univariate and multivariate analysis was also performed separately for the patients who received R0 resection (Supplementary Table S3 and Figure 4B). The results indicated that pN3b and signet ring cell carcinoma were significant risk factors for peritoneal recurrence of Borrmann type IV GC.

## Discussion

The incidence of Borrmann type IV gastric cancer has a large deviation in previous reports, ranging from 8 to 20% (3, 4, 9). In recent years, many relevant studies have shown that Borrmann type IV gastric cancer was characterized as higher female/male ratio, poorer differentiation, higher risk of serosal infiltration, lymph node metastasis and peritoneal metastasis, and poor prognosis (4, 9). Lee et al. reported that Borrmann type IV gastric cancer is an independent risk factor for peritoneal recurrence (15). In this study, we mainly included patients who underwent gastrectomy, and the incidence of Borrmann type IV GC was about 7.8%. Due to the aggressiveness of Borrmann type IV GC, many patients had already lost the opportunity for surgical treatment when they were diagnosed, so our incidence rate was slightly lower than that shown in relevant reports. Many studies have reported the prognostic factors of Borrmann type IV GC. For instance, Yamashita et al. suggested that elder age, T category, N category, peritoneal dissemination, CY1/CYX and margin status are prognostic factors of Borrmann type IV GC, in which elder age, T category and peritoneal dissemination are independent prognostic factors (17). Univariate analysis by Yook et al. found that tumor location, occupied region, invasion depth, lymph node metastasis and pTNM stage were correlated with the prognosis of Borrmann type IV gastric cancer after radical surgery (18). The multivariate analysis indicated that only tumor location and pTNM stage were independent factors affecting the prognosis of Borrmann type IV gastric cancer after radical surgery. In this study, a multivariate analysis of 159 patients showed that pN3b (*P* = 0.03), along with residual tumor, was a significant independent prognostic factor of Borrmann type IV GC, which was basically consistent with previous reports. The treatment options for Borrmann type IV GC are difficult and controversial because of its high incidence of peritoneal metastasis and poor prognosis. Early detection of Borrmann IV gastric cancer by endoscope remain difficult due to the diffuse invasion of cancer cells to the mucosa lamina propria and no obvious ulcer or mucosal surface uplift (3, 8). At diagnosis, cancer cells often penetrate the serous membrane and have lymph node metastasis. Curative resection (R0 resection) is critical for treatment of this GC subtype (4, 5).

Previous reports have shown that nearly 20% of patients with GC were diagnosed with PM before or during surgery, and about 50% of patients developed PM after radical surgery (19, 20). Patients with PM had a poor prognosis with a median OS of less than 2 years (19, 21). PM eventually lead to refractory ascites, intestinal obstruction and cachexia, which are the main causes of death of gastric cancer (20). Many clinical studies have investigated the risk factors of PM in GC. Huang BJ et al. suggested that patients with Borrmann III/IV and N3 should be closely followed to detect peritoneal metastasis (22). Several other studies have shown a significantly close relationship between lymph node metastasis and PM (23, 24). Among the characteristics of Borrmann type IV GC, it is worth noting that the incidence of PM is much higher than other types (9). This is also one of the main reasons for the poor prognosis of this GC subtype. It is noteworthy that the tendency of Borrmann type IV to develop PM was concurrent with a lower risk of liver metastasis, suggesting a specific pattern of metastasis (9). Consistently, among 49 who developed distant metastasis in our cohort, 43 (87.8%) developed PM but only 3 (6.1%) developed liver metastasis. Otsuji E et al. considered lymph node metastasis as an independent risk factor for PM of Borrmann type IV GC in 1999 in a cohort of 150 patients (25). In addition, Dong RZ et al. concluded that extracapsular lymph node spread (ECS) is an independent prognostic factor and an adverse factor for PM in patients with Borrmann type IV gastric cancer with radical resection (3). Otherwise, there are few studies focus on the risk factors associated with peritoneal metastasis of Borrmann type IV GC. In this study, we found that regional lymph node metastasis and tumor size were the variables that independently correlated with PM. Furthermore, pN3b and signet ring cell carcinoma were the independent predictor of PM in patients who had received R0 resection (peritoneal recurrence).

Among several clinicopathologic factors, the tumor size clinically served as a simple predictor of tumor progression (26). Previous study reported that tumor size was strongly correlated with the depth of invasion, degree of lymph node metastasis, and stage of the disease. Saito et al. (27) reported that tumor size might be a good indicator in the prediction of recurrence site as well as serve as a simple predictor of survival of patients with gastric cancer. These results indicate that tumor size provides important information about the malignant potential of tumors. Patients with larger tumors may need more aggressive treatment and more frequent postoperative re-examination.

Gastric signet ring cell carcinoma (GSRC) is a typical diffuse infiltrating gastric cancer with low differentiation, strong invasiveness and poor prognosis (28–31). Most of GSRCs were Borrmann type III and IV gastric cancer (30). Previous studies [6,8] found that GSRC showed a higher incidence of peritoneal metastasis (30, 32) and was an independent factor affecting lymph node metastasis (32), while the latter was an independent prognostic factor of advanced GC (32). These results suggest that one of the reasons for the poor prognosis of gastric SRCC is the tendency of lymph node metastasis. In our study, GSRC was an independent risk factor of peritoneal recurrence which suggested that carefully follow-up examinations and more aggressive treatment may be necessary for Borrmann type IV GC with SRC after surgery.

Regional lymph node metastasis played an important role in predicting prognosis and peritoneal recurrence in our study, especially pN3b. The American Joint Committee on Cancer (AJCC) TNM staging system is currently recognized as the best malignant tumor staging system in the world, and its latest 8th edition was published in October 2016, replacing the 7th edition since 2009 (33–35). Although the 7th edition divided N3 into N3a and N3b, it did not impact the TNM staging of GC. However, a study of over 25,000 GC patients from 15 countries found that N3a and N3b two subgroups of patients with significant differences of its survival. Subsequently, in the updated 8th edition of TNM staging system, N3a and N3b largely impact the tumor staging (33, 35). The new grading system has been validated in national databases to verify its predictive power and accuracy (36–39). Some studies have identified that patients with pN3a and pN3b presented distinct survival outcomes (34, 40), which is consistent with our results (Table 1 and Supplementary Table S4). Yonemura Y. et al. reported trans-lymphatic metastasis as one of the PM formation concepts (41). In our study, univariate Cox analysis showed that pN3b was significantly different from other N stages in predicting prognosis and peritoneal recurrence in Borrmann type IV GC (Figures 3A,B). This might be because more tumor cells exist in patients with lymph node metastasis, spreading through the lymphatic system, also patients with pN3b indicates a more locally advanced disease, which might thus be accompanied by a higher incidence of transperitoneal spread. Therefore, we conclude that for patients with Borrmann type IV GC, lymph node metastasis greater than 15 is a better cut-off value to predict poor prognosis and high incidence of peritoneal recurrence. Thus, we could make more effort for these patients in order to improve their prognosis. Firstly, routine gastroscopy physical examination and early detection of cancer without lymph node metastasis may be effective means of prevention. Secondly, for patients with N3b indicated by preoperative imaging examination, it is more necessary to perform neoadjuvant therapy and more active treatment measures including prophylactic intraperitoneal chemotherapy to achieve the purpose of a better prognosis. Last but not least, more intensive follow-up for N3b patients may be meaningful for early detection and intervention of metachronous peritoneal metastasis to improve the outcomes of patients with Borrmann type IV GC.

Whereas, there are some limitations of our study. This study is a retrospective and single-institution study and only those patients who referred to our hospital for surgery were enrolled. A very few patients had less than 16 lymph nodes dissected due to the lack of standardization of surgical methods in the early years. For Borrmann I–III GC, we lacked some information on recurrence, making it difficult for us to make a more accurate comparison. These limitations can lead to biases that may affect the accurate evaluation. A multicenter, prospective study is needed to validate these results in a larger population in future. Also, neoadjuvant therapy plays an important role in the treatment of Borrmann type IV GC due to its malignant biological behavior. However, pN stage is inferred from pathological findings which might not be disadvantageous in choosing treatment options.

## Conclusion

In summary, retrospective analysis of clinicopathological factors in Borrmann type IV GC revealed that lymph node metastasis, specifically pN3b, as an independent prognostic factor. Lymph node status and tumor size were identified as independent predictors of PM. Importantly, pN3b is an important predictive factor for worse prognosis and peritoneal recurrence after radical surgery in patients with Borrmann type IV GC.

## Data Availability

The raw data supporting the conclusions of this article will be made available by the authors, without undue reservation.
