# Supplementary material for: PN3b as an independent risk factor for poor prognosis and peritoneal recurrence in Borrmann type IV gastric cancer: A retrospective cohort study
Source: Front Surg. 2022 Nov 10;9:986696. doi: 10.3389/fsurg.2022.986696 (PMC9684711; doi:10.3389/fsurg.2022.986696)
Supplement: Supplementary file 2 [file Datasheet1.pdf]

Supplementary Material

Supplementary Figures

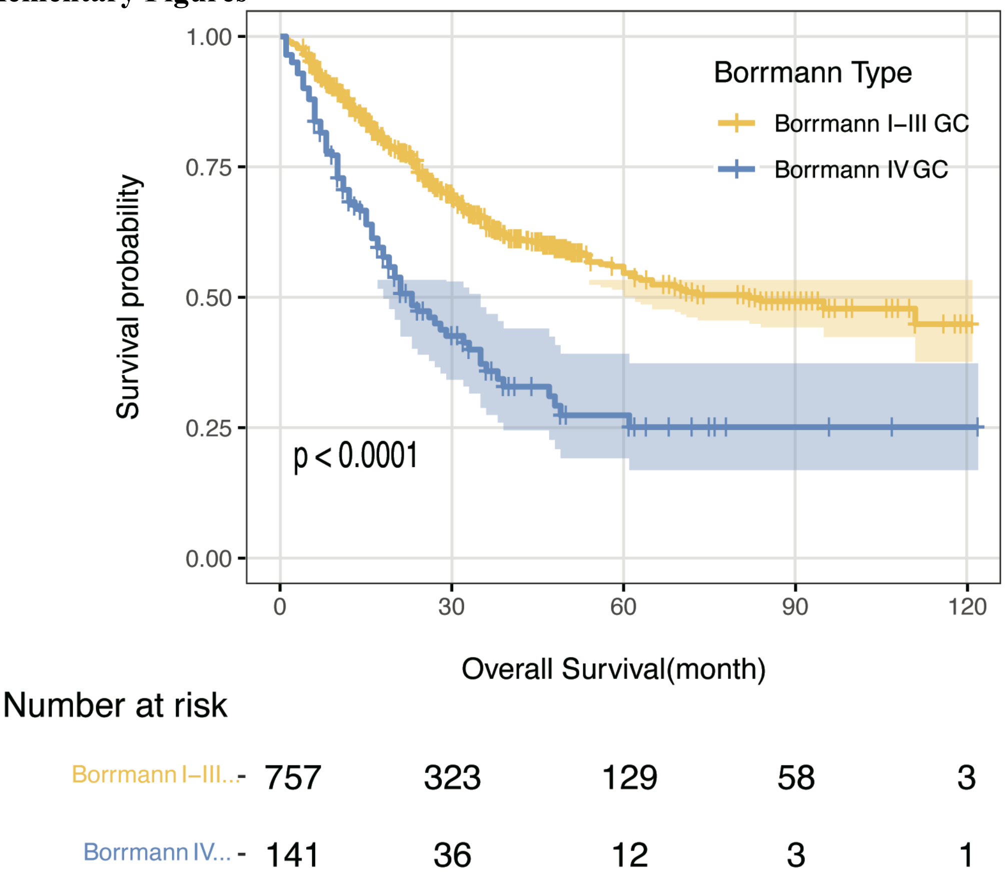

Supplementary Figure 1. Kaplan-Meier curve of overall survival (OS) according to Borrmann type.

		Univariable analysis		Multivariable analysis	
**Variable**		**Median OS (m)**	***P* value**	**Hazard ratio(95%CI)**	***P* value**
**Gender**	Female	54		Reference	
	Male	63	0.891	0.99 (0.75, 1.32)	0.968
**Age**	< 60	111		Reference	
	≥ 60	50	0.02	1.48 (1.13, 1.93)	0.004
**Residual Tumor**	R0	71		Reference	
	R1	16		1.75 (1.06, 2.89)	0.029
	R2	6	<0.001	3.34 (2.05, 5.44)	<0.001
**SRC**	No	69		Reference	
	Yes	36	0.025	1.05 (0.75, 1.46)	0.789
**TNM Stage**	I	-		Reference	
	II	-		3.48 (1.08, 11.27)	0.037
	III	38		9.52 (3.03, 29.88)	<0.001
	IV	8	<0.001	23.65 (7.16, 78.08)	<0.001
**Borramnn Type**	Borrmann I–III	82		Reference	
	Borrmann IV	23	<0.001	1.72 (1.25, 2.36)	<0.001
**CEA**	≤ 5.00ng/ml	95		Reference	
	> 5.00ng/ml	32	<0.001	1.43 (1.11, 1.86)	0.006
**CA199**	≤ 37.00U/ml	95		Reference	
	> 37.00U/ml	33.5	<0.001	1.15 (0.87, 1.51)	0.317

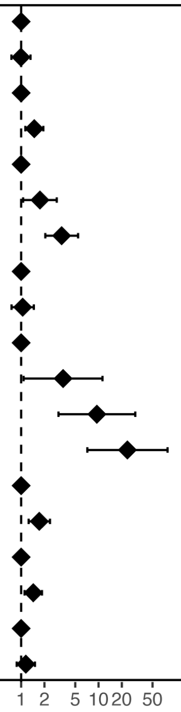

Supplementary Figure 2. Forest plot displaying the results of hazard ratio for overall survival of 920 advanced gastric cancer patients. (SRC, signet ring cell)

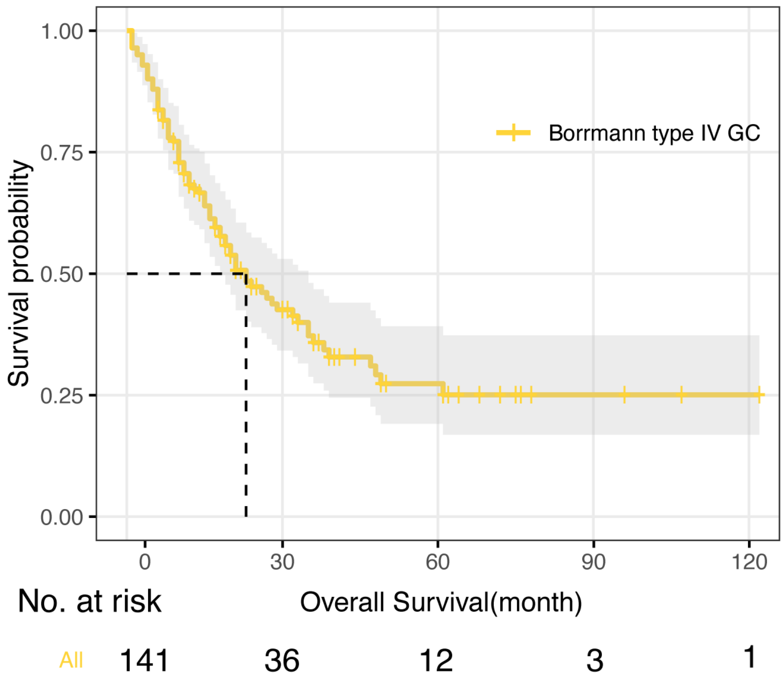

Supplementary Figure 3. Survival curve of Borrmann type IV GC patients (n =159. Five-year survival rate 25.1%).

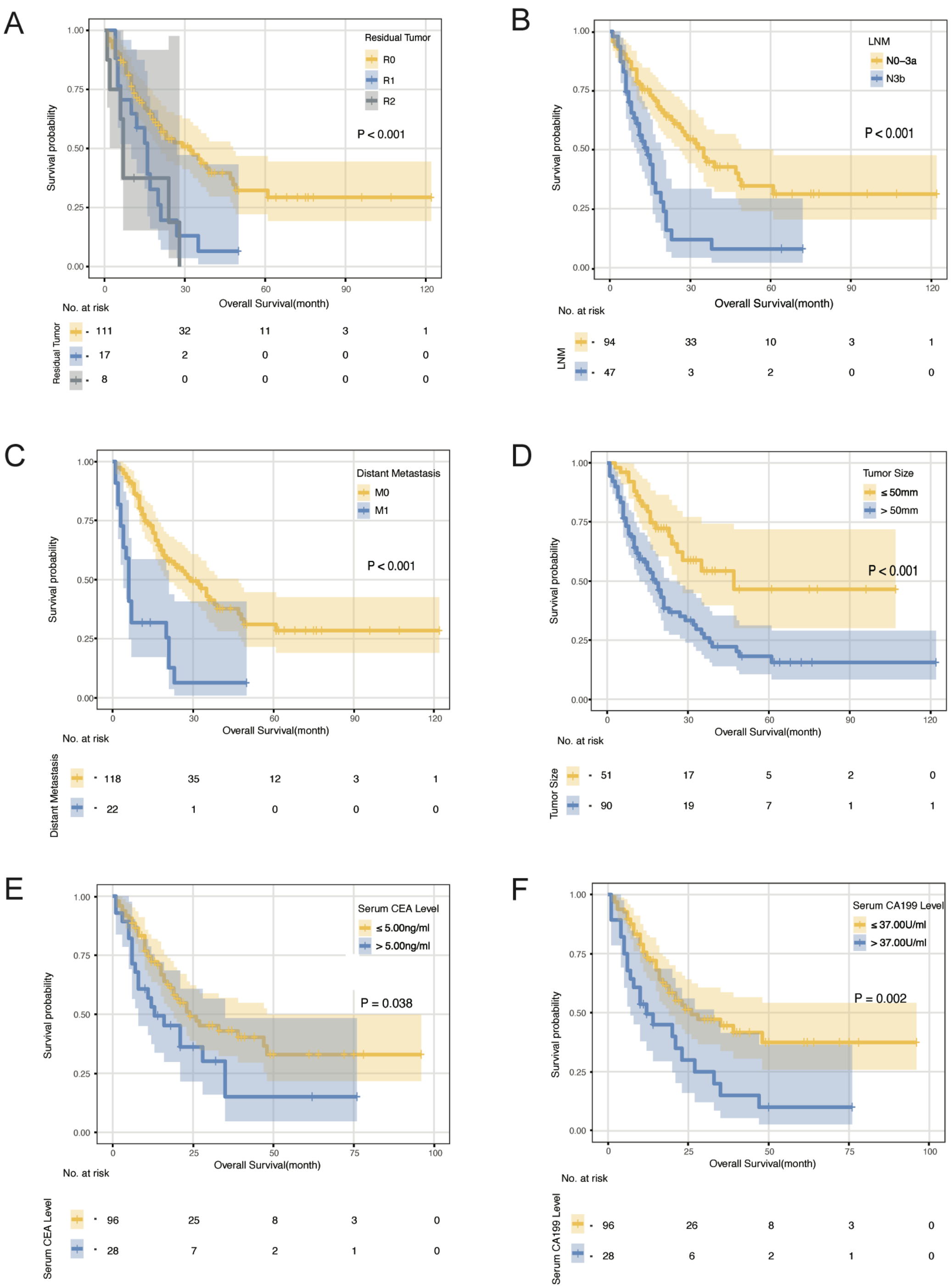

Supplementary Figure 4. Kaplan-Meier curves of overall survival (OS) in Borrmann type IV gastric cancer. (A-F): Kaplan-Meier curves of overall survival (OS) according to residual tumor, lymph node metastasis, distant metastasis, tumor size, level of serum CEA and CA19-9 in Borrmann type IV gastric cancer. (LNM: lymph node metastasis).

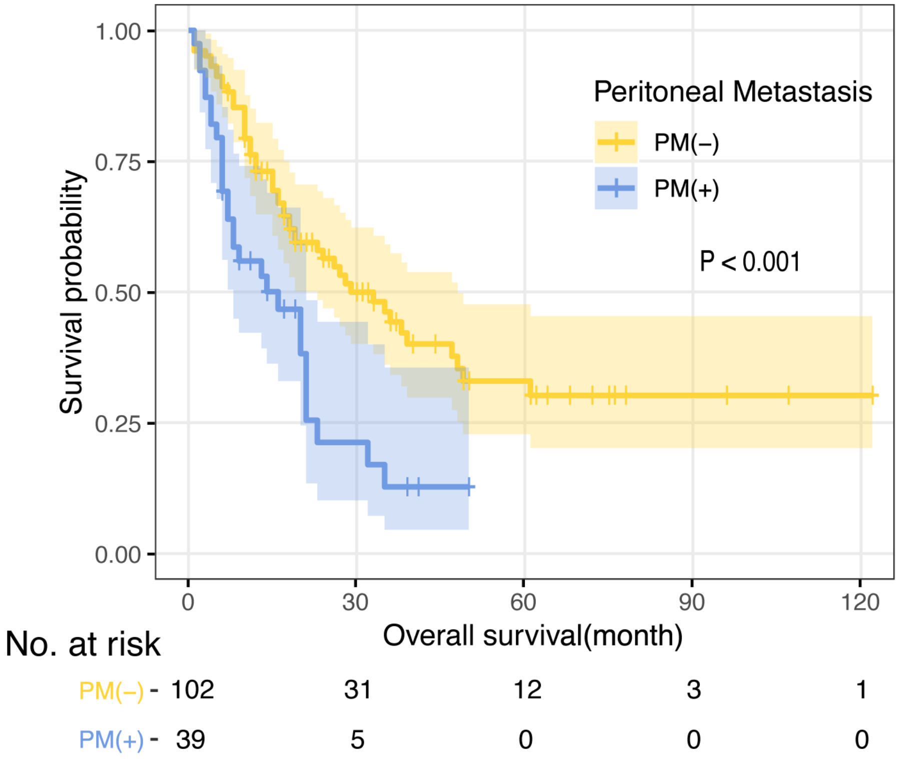

Supplementary Figure 5. Kaplan-Meier curves of overall survival (OS) according to PM in Borrmann type IV gastric cancer.
